# Bayesian Inference of Vocal Fold Material Properties from Glottal Area Waveforms Using a 2D Finite Element Model

**DOI:** 10.3390/app9132735

**Published:** 2019-07-06

**Authors:** Paul J. Hadwin, Mohsen Motie-Shirazi, Byron D. Erath, Sean D. Peterson

**Affiliations:** 1 Department of Mechanical and Mechatronics Engineering, University of Waterloo, Waterloo, ON N2L 3G1, Canada; 2 Department of Mechanical and Aeronautical Engineering, Clarkson University, Potsdam, NY 13699, USA

**Keywords:** patient-specific modeling, silicone vocal fold models, Bayesian inverse analysis, finite element analysis

## Abstract

Bayesian estimation has been previously demonstrated as a viable method for developing subject-specific vocal fold models from observations of the glottal area waveform. These prior efforts, however, have been restricted to lumped-element fitting models and synthetic observation data. The indirect relationship between the lumped-element parameters and physical tissue properties renders extracting the latter from the former difficult. Herein we propose a finite element fitting model, which treats the vocal folds as a viscoelastic deformable body comprised of three layers. Using the glottal area waveforms generated by self-oscillating silicone vocal folds we directly estimate the elastic moduli, density, and other material properties of the silicone folds using a Bayesian importance sampling approach. Estimated material properties agree with the “ground truth” experimental values to within 3% for most parameters. By considering cases with varying subglottal pressure and medial compression we demonstrate that the finite element model coupled with Bayesian estimation is sufficiently sensitive to distinguish between experimental configurations. Additional information not available experimentally, namely, contact pressures, are extracted from the developed finite element models. The contact pressures are found to increase with medial compression and subglottal pressure, in agreement with expectation.

## 1. Introduction

Numerical models have long been employed to better understand the complex physics involved in human phonation. For example, reduced-order and finite element numerical models of the vocal folds (VFs) can self-oscillate in a manner representative of actual VF kinematics during sustained vowels [1], pitch glides [2], and, in a few cases, running speech [3]. Such models have explored a wide range of phenomena relevant to normal and pathological phonation, including the impact of a posterior glottal gap [4], the ventricular folds [5], phonation onset pressure [6], and the efficacy of various compensation mechanisms for vocal hyperfunction [7,8].

Whereas the majority of modeling efforts in speech, to date, have employed models with general population-based parameters to uncover the universal physical underpinnings of human phonation, a few research teams have begun to explore the development of subject-specific numerical models for phonation [9–13]. The dynamics of the VFs are sensitive to a variety of factors, including subglottal pressure [14], laryngeal muscle activation [15], and a posterior glottal gap [4], to name a few. These factors, which can play a role in vocal hyperfunction [16] and other pathologies [17–20], can be a challenge to observe clinically. Subject-specific models, on the other hand, are constructed based on measurements of the subject through less challenging media, such as high speed videoendoscopy (HSV) [9,11,21] and offer the potential to elucidate clinically opaque features and parameters, such as VF contact pressures.

In general, development of a subject-specific model entails estimation of numerical parameters such that the model behavior “matches” some observed data from the individual of interest, such as VF kinematics from HSV [9,11,21]. Specifically, numerical model parameters are sought such that some model output(s) (e.g., the VF kinematics) best match the equivalent observation data from the subject. This problem is ill-posed, thus necessitating inverse analysis techniques to determine the model parameters [22]. To date, optimization [9,22], machine learning [23], and Bayesian [10,11,21] frameworks have been developed to estimate VF model parameters.

Optimization-based approaches define a cost functional and employ optimization techniques to determine the parameter values that minimize the functional. Döllinger et al. [22] was the first to successfully use this approach, employing the Nelder-Mead algorithm to determine the vibrating masses, spring stiffnesses, and subglottal pressure of a two mass VF model by minimizing the least-squares error between specific Fourier coefficients of the measured VF trajectories and the simulated trajectories. Genetic algorithms were subsequently employed to determine parameters of a two mass model that best reproduced the trajectories from patients suffering from unilateral VF paralysis [16]. Inverse procedures have also been used to classify disordered versus healthy VF oscillations. Wurzbacher et al. [24] employed simulated annealing to minimize the Euclidean distance between the model and experimental vocal fold trajectories to distinguish between normal and dysphonic subjects during sustained vowel phonation and a pitch glide. Very recently, deep learning tools have been employed for subject-specific modeling [23]. A long short-term memory network, trained on simulated data, was used to estimate the subglottal pressure from ex vivo recordings of porcine vocal folds. They were able to produce accurate estimates of the subglottal pressure with very low online computational costs.

The vast majority of these subject-specific VF modeling efforts, and all of those mentioned above, have used lumped-element representations of the VFs and employed inverse methods to determine the reduced-order parameters. Of noted exception are the efforts by Xue et al. [12] and Chang et al. [13] that employ finite element model representations of the VFs. Xue et al. used computed tomography to reconstruct the larynx of a subject in a computational domain. The external geometry of the VFs was obtained from the scan, whereas the VF layers and material properties were assumed from population-based histological data. Chang et al. constructed a laryngeal model of a rabbit from magnetic resonance images. Two VF layers were observed in the scans, leading to a body-cover-type construction wherein the densities and Poisson ratios were assumed a priori. The elastic moduli of the layers were determined by an informal optimization that attempted to match the maximum glottal width and fundamental frequency of the model with HSV measurements.

In contrast to optimization-based and machine learning methods that treat estimated model parameters as deterministic, the Bayesian framework treats all parameters and measurements as random variables. Such a framework has the benefit of elucidating the propagation of measurement uncertainties to the model parameters and outputs. Uncertainty estimates are powerful as they provide additional information about the fidelity of the model outputs, which are expected to be of value for clinical decisions. Cataldo et al. [10] introduced the Bayesian framework to estimate stationary (non-time-varying) parameters of a two mass VF model using an importance sampling approach. This work was extended to estimate non-stationary parameters of a three mass body-cover model using both a particle filter [11] and an extended Kalman filter [21]. The Bayesian framework has, thus far, been demonstrated using simulated observation data from a reduced-order VF model, wherein the ground truth was known a priori, to estimate the parameters of a different reduced-order VF fitting model. The similarity between the generation and fitting models enabled direct assessment of the performance of the Bayesian framework. Herein we build on the Bayesian inference framework for developing subject-specific VF models by: (i) considering real HSV recordings of self-oscillating silicone VFs as the observation data; and, for the first time, (ii) employing a two-dimensional (2D) finite element (FE) model of the VFs as the fitting model. These two considerations address several limitations in prior Bayesian estimation-based inference studies applied to voiced speech, while simultaneously extending the methodology to more complex VF representations. Specifically, actual experimental data, with its inherent uncertainties, are used as the observations. Furthermore, by employing a FE representation of the VFs, the estimated FE parameters have direct relation to the material properties of the silicone VF models, reducing the layer of abstraction associated with reduced-order models. By using silicone VFs to generate the observation data, however, the “ground truth” is still available to assess the performance (estimated properties and their uncertainties) of the estimation procedure. Lastly, the use of experimental data provides a more realistic test bed due to the dynamical differences between the data source and the fitting model.

We consider two different VF base configurations, with and without medial compression, as well as multiple subglottal pressures. Exploring medial compression offers insights into how FE models and Bayesian estimation can incorporate VF posturing for subject-specific models, while varying subglottal pressure affords a mechanism for evaluating how well Bayesian inference, when accounting for measurement uncertainty, can differentiate between phonation conditions.

The paper is structured as follows: Section 2 discusses the silicone VFs and experimental conditions used during collection of the data sets and handling of the HSV. Respectively, Sections 3 and 4 briefly discuss the mathematical model and estimation procedure used in the analysis. Section 5 presents the results of the estimates and provides discussion of the findings. Additionally, this section examines how the numerical parameters, including the triangulation density and step size in time, effect the subject-specific estimates. The manuscripts concludes with Section 6.

## 2. Experimental Setup and Data Collection

We employ the measured kinematics of silicone VFs undergoing self-sustained oscillations as a proxy for clinical HSV recordings. Silicone VFs have been shown to exhibit similar kinematics to real VFs [25], and thus serve as a reasonable test platform for exploring the viability of Bayesian estimation for producing subject-specific models from clinical data. Furthermore, the known histology and material properties of the silicone VFs provide “ground truth” data with which to compare property estimates. This section discusses the details of the silicone VF experiments, including the manufacturing process used to develop the silicone VFs, the experimental procedures used to capture the HSV, and extraction of the glottal area waveform (GAW) from the HSV, which will serve as the observation data in the inverse analysis.

### 2.1. Silicone Vocal Fold Models

**Figure 1.**
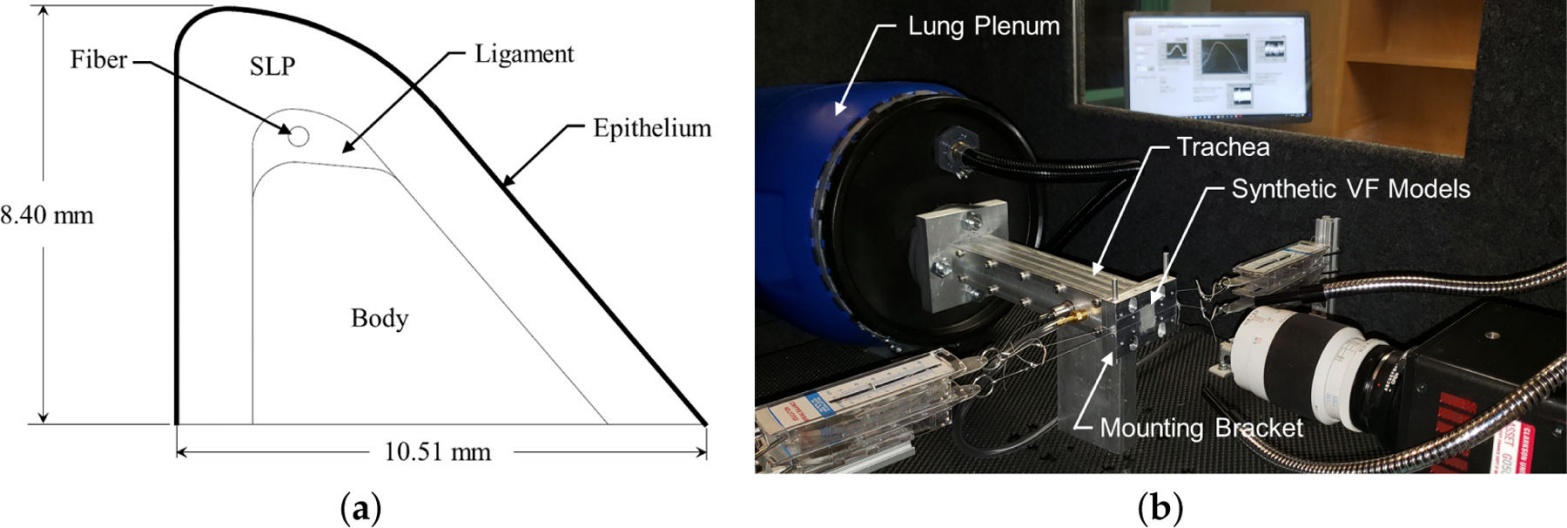
(**a**) Model of the geometry of the silicone vocal folds; and (**b**) image of the experimental flow facility.

Silicone VFs are manufactured to reflect the physiological VF structure. The geometry, shown in Figure 1a, is based on the M5 geometry [25] and comprises four layers to reflect the layered VF structure; namely, the body, ligament, superficial lamina propria (SLP), and epithelium. Additionally, a fiber is included in the middle of the ligament layer to control anterior-posterior stiffness. Each VF has an anterior-posterior length of 17.00 mm, inferior-superior depth of 10.51 mm, and a medial-lateral height of 8.40 mm.

The manufacturing process of each silicone VF follows previous approaches [25], where the specific ratios of part A, B, and thinner used to manufacture each layer of the VFs, and the approximate resulting value of the elastic modulus, can be found in Table 1. The same silicone VF models were used for all tests.

### 2.2. Laryngeal Flow Facility

The synthetic VFs were mounted in a custom laryngeal flow facility driven by compressed air (~550 kPa) that was regulated down to 17.0 kPa via a Siemens 40–2 pressure regulator. The flow then passed through a Dwyer RMC 103-SSV flow meter that measured the volumetric flow rate and further regulated the flow before entering the vocal tract test facility shown in Figure 1b. The facility was comprised of a model lung plenum, which consisted of a 0.03 m^3^ cylindrical chamber that was acoustically treated on the inside to reduce acoustic reflections. The plenum exhausted to a square tube, representing the trachea, with a cross-sectional area of 4.94 cm^2^ and 48.0 cm in length. The subglottal pressure was measured with a flush-mounted Kulite ET-3DC pressure transducer in the wall of the trachea tube, 3.8 cm upstream of the glottal exit.

Two square mounting brackets that housed the synthetic VF models were attached at the exit plate of the tracheal tube. An inset within the tracheal tube transitioned the interior dimensions of the square tracheal tube down to 1.7 cm × 1.7 cm over a length of 2.5 cm. While the medial-lateral dimension of each VF is 8.4 mm, an inset depth of 7.6 mm was machined into each opposing mounting bracket to hold the VFs. In this manner, the medial surface extended above the contacting surface of each bracket by 0.8 mm, such that when the opposing VFs were brought into contact the amount of medial compression could be adjusted.

Two data sets were collected. The first positioned the VFs in a natural, uncompressed position such that there was no medial compression. The second incorporated a 0.8 mm shim between the opposing brackets when the medial surfaces were positioned in contact. This resulted in each VF being compressed by 0.4 mm in the medial-lateral direction. Anterior–posterior tensioning was applied in all cases by pulling on the string embedded in the models with a constant force of 0.3 N. Note that in this study there was no supraglottal tract, see Figure 1b.

High-speed video of the VF motion was acquired using an 8-bit IDT MotionPro X3 PLUS camera equipped with an Elicar V-HQ Macro 90 mm lens. In this study, an array size of 484 × 504 pixels was used with a frame rate of 2000 fps. The spatial resolution of the videos was 24.4 pixels/mm, corresponding to a physical area for each pixel of 1.680 × 10^−3^ mm^2^.

For the case without medial compression, the VFs were driven with a subglottal pressure of 1.00 kPa. With medial compression, data were collected for subglottal pressures of 0.91, 1.00, 1.09, and 1.18 kPa, yielding average flow rates of 236, 260, 291, and 307 mL/s, respectively. HSV was recorded for 1 s in each configuration, from which a 300 ms segment was used to extract the GAW using segmentation [26].

## 3. Finite Element Model

Estimates of the silicone VF material properties were computed by matching the simulated motion with the kinematics captured by the HSV. To simulate the dynamics we employed a FE representation of the silicone VFs based upon the model developed by Alipour et al. [27], where the displacement field of a transversely isotropic linear medium is approximated using a FE basis defined by piece-wise linear functions over triangular elements. The FE formulation, which is functionally equivalent to Alipour et al. [27], is briefly described in this section.

### 3.1. Mathematical Model

Small deformations of the silicone VFs can be modeled using a displacement field **u**, which describes the displacement of two linear transversely isotropic elastic bodies [27]. The VFs were oriented such that the medial-lateral direction defines the *x*-axis, the *y*-axis is in the anterior-posterior direction, and the *z*-axis is positive in the superior direction. As a simplification, the dynamics of the two VFs are treated as symmetric and displacement is treated as uniform along the length of the VFs; as such, the displacement field does not vary along the *y*-axis, i.e.,

(1) $$u=u(x,\;t)=u_{x}(x,\;t)\hat{i}+u_{z}(x,\;t)\hat{j},$$

where **x** = [*x*, *z*]^T^ is a vector of planar spatial coordinates. This reduction of physical dimension greatly reduces the computational cost of the FE model.

Given a deformation with displacement field **u** and an arbitrary variation **u**^*δ*^, the virtual work principle gives [28]

(2) $$\int_{\Omega }{\left(u^{\delta }\right)}^{\text{T}}\rho (x)ü\;\text{d}V+\int_{\Omega }\sigma (u):\varepsilon \left(u^{\delta }\right)\;\text{d}V=\int_{\Gamma }{\left(u^{\delta }\right)}^{\text{T}}f_{\text{s}}\;\text{d}S,$$

where Ω and Γ are the 2D VF domain and its boundary, respectively, *ρ* is the material density, ***σ*** and ***ε*** are the stress and strain tensors, and **f**_s_ is the surface force, in this case arising from aerodynamics.

Since the vocal folds are modeled as isotropic in the *xz*-plane, Hooke’s law gives [28]

(3) $$\sigma (u):\varepsilon \left(u^{\delta }\right)=\tilde{\sigma }{\left(u\right)}^{\text{T}}\tilde{\varepsilon }\left(u^{\delta }\right)=\tilde{\varepsilon }{\left(u^{\delta }\right)}^{\text{T}}\tilde{\sigma }(u)=\tilde{\varepsilon }{\left(u^{\delta }\right)}^{\text{T}}C\tilde{\varepsilon }(u),$$

where $\tilde{\varepsilon }(u)={\left[\frac{\partial u_{x}}{\partial x},\frac{\partial u_{z}}{\partial z},\frac{\partial u_{z}}{\partial x}+\frac{\partial u_{x}}{\partial z}\right]}^{\text{T}}$ and **C** is a positive definite matrix given by

(4) $$C(E,v)=\left(\begin{array}{ccc} \lambda +2\mu & \lambda & 0\\ \lambda & \lambda +2\mu & 0\\ 0 & 0 & \mu \end{array}\right)=\mu (E,v)\left(\begin{array}{ccc} \frac{2(1-v)}{1-2v} & \frac{2v}{\left(1-2v\right)} & 0\\ \frac{2v}{\left(1-2v\right)} & \frac{2(1-v)}{1-2v} & 0\\ 0 & 0 & 1 \end{array}\right).$$

Here *λ* and *μ* are the Lamé parameters that are related to the Young’s modulus *E* and Poisson’s ratio *ν* via

(5) $$\mu (E,\;v)=\frac{E}{2(1+v)},\;\lambda (E,\;v)=\frac{vE}{\left(1+v)(1-2v\right)}.$$

Note that for **C**(*E*, *ν*) to be positive definite requires *E* > 0 and −1 < *ν* < 1/2.

Finally, following Alipour et al. [27], the viscous damping experienced by the VFs is modeled by replacing the shear modulus, *μ*(*E*, *ν*), with *μ* + *η*d/d*t*, where *η* is viscosity.

### 3.2. Finite Element Approximation

The displacement field is approximated with a linear combination of piece-wise linear basis functions ${\left\{\phi_{i}\right\}}_{i=1}^{N}$, given by

(6) $$u(x,t)\approx \left(\begin{array}{l} \sum_{i=1}^{N}\alpha_{i}(t)\phi_{i}(x)\\ \sum_{i=1}^{N}\beta_{i}(t)\phi_{i}(x) \end{array}\right)$$

where each *ϕ*_*i*_(**x**) is defined over a set of connected elements that approximate the geometry of the VFs. The FE approximation is then found by substituting this approximation into Equation (2), giving

(7) $$M\ddot{\theta }+D\dot{\theta }+K\theta =F,$$

where

(8) $$M=\int_{\Omega }\rho \Phi^{\text{T}}\Phi \;\text{d}V,\;D=\int_{\Omega }\eta \Phi_{d}^{\text{T}}S\Phi_{d}\;\text{d}V,\;K=\int_{\Omega }\Phi_{d}^{\text{T}}C\Phi_{d}\;\text{d}V,\;\text{ and }\;F=\int_{\Gamma }\Phi^{\text{T}}f_{\text{s}}\;\text{d}S.$$

Here,

(9) $$\theta (t)={\left[\alpha_{1}(t),\ldots \alpha_{N}(t),\beta_{1}(t),\ldots \beta_{N}(t)\right]}^{\text{T}},$$

(10) $$\Phi (x)=\left(\begin{array}{cccccc} \phi_{1}(x) & \cdots & \phi_{N}(x) & 0 & \ldots & 0\\ 0 & \ldots & 0 & \phi_{1}(x) & \ldots & \phi_{N}(x) \end{array}\right),$$

(11) $$\Phi_{d}(x)=\left(\begin{array}{cccccc} \partial_{x}\phi_{1}(x) & \ldots & \partial_{x}\phi_{N}(x) & 0 & \ldots & 0\\ 0 & \cdots & 0 & \partial_{z}\phi_{1}(x) & \ldots & \partial_{z}\phi_{N}(x)\\ \partial_{z}\phi_{1}(x) & \ldots & \partial_{z}\phi_{N}(x) & \partial_{x}\phi_{1}(x) & \ldots & \partial_{x}\phi_{N}(x) \end{array}\right),$$

and integration is interpreted element wise. The computation of **M**, **D**, and **K** now follow the standard finite element construction [28].

The boundary of the VFs was split into two sections: (*i*) a section where external forces are applied, denoted Γ_free_, and (*ii*) a section where the nodes do not move, which represents the walls of the trachea, denoted Γ_fixed_. The vector **F** is the nodal force vector and is the result of the transverse surface force. In this work aerodynamic pressure is treated as the only stress leading to an external force on the surface nodes. The pressure at each node along the free surface is modeled using a one-dimensional Bernoulli flow model, which gives

(12) $$p(s,t)=\{\begin{array}{cc} p_{\text{sub}}-\left(p_{\text{sub}}-p_{\text{sup}}\right){\left(\frac{A_{\text{sep}}(t)}{A(s,t)}\right)}^{2}, & A(s,t)<A_{\text{sep}}\\ p_{\text{sub}}, & A(s,t)\geq A_{\text{sep}} \end{array}$$

where *s* ∈ Γ_free_, *p*_sub_ and *p*_sup_ are the subglottal and supraglottal pressures, respectively, *A*_min_(*t*) is the minimum glottal area at time *t*, *A*(*s*, *t*) is the area at location *s* at time *t*, and *A*_sep_ = 1.3*A*_min_(*t*) is the glottal area at the location of flow separation [29,30].

Vocal fold collision is modeled by restricting the *x*-coordinate of each node from crossing the midline. Should a time step result in a node crossing the midline, its *x*-coordinate is forced to the midline. The force required to restrict the node to the midline is directly the contact force. Contact pressure can be obtained by dividing by the edge area (distance between the the node and its neighbor).

Finally, time integration was achieved through the finite differencing scheme set out by Alipour et al. [27]. The time integration gives the displacement field over time, yielding a position time series for each node in the finite element mesh. From this the glottal width, equivalent to what can be observed with HSV; *W*_gl_ is given by *W*_gl_(*t*) = 2 min{*x*_1_(*t*), *x*_2_(*t*), …, *x*_*N*_(*t*)}, where *N* is the number of nodes, and *x*_*i*_(*t*) represents the *x*-coordinate position time series the of the *i*^*th*^ node. Thus the simulated glottal area, *A*_s_, can be computed as *A*_s_(*t*) = *ℓ*_gl_ × *W*_gl_(*t*), where *ℓ*_gl_ is the length of the glottis, a constant in the two-dimensional approximation employed herein.

## 4. Estimation of VF Material Properties

The FE model embeds the material properties (e.g., *E*, *ρ*, etc.) that we aim to directly estimate via Bayesian inference. This section details the parameterization of the FE model and the Bayesian inference procedure employed herein.

### 4.1. Parameterization of the FE Model

**Figure 2.**
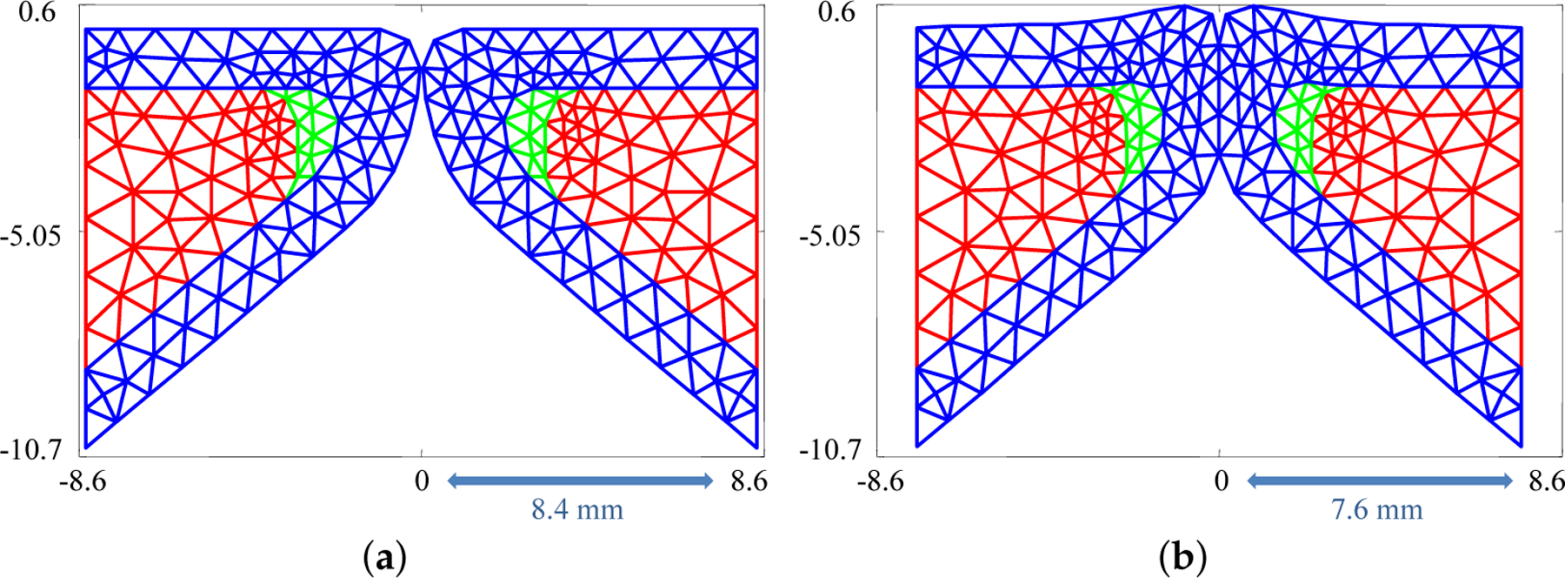
(**a**) Finite element triangulation used to simulate the silicone vocal folds; and (**b**) mesh deformation occurring as a result of medial compression. Red region: body; green region: ligament; and blue region: cover.

The silicone VFs were numerically modeled with three layers: the body, cover (which combines the SLP and epithelium), and ligament. Employing the same dimensions as the silicone VFs (see Section 2.1), a FE representation was generated comprising 205 triangular elements and 120 nodes, see Figure 2a. The sensitivity of the results to the triangularization is discussed in Section 5.3.

All material properties are modeled as uniform across all layers except for the Young’s modulus, which differs from layer to layer, but is constant within a layer. As a result, the FE model is parameterized by: *ρ*, *η*, *E* (for each layer), *ν*, and *p*_sub_ and *p*_sup_. The density, viscosity, Young’s modulus for each layer, and subglottal pressure were estimated while all other parameters were treated as fixed and known. Specifically, we assumed *ν* = 0.4995 [31] and *p*_sup_ = 0 Pa. This parameterization results in low dimensionality for the estimation problem while still employing a high degree-of-freedom VF model.

As discussed in Section 2.2, medial compression of the silicone VFs is considered. Medial compression pre-stresses the VFs introducing more initial potential energy into the system. The compression is modeled via an initial position parameter, *x*_0_, which represents the maximum value that the *x* coordinate of any node can attain. As a result, having *x*_0_ = 8.4 mm indicates no medial compression and any value 0 ≤ *x*_0_ < 8.4 mm indicates the presences of medial compression, as shown in Figure 2. The initial displacement of each node ***θ***_0_ in cases involving medial compression was calculated from Equation (7) with time derivatives set to zero. This equation was solved iteratively by adjusting the *x* coordinate of the nodes in steps of 10^−4^ mm while enforcing that no node cross the midline.

The FE simulations used a time step of *h* = 0.05 ms [27], which is equivalent to a frame rate of 20,000 fps. Since the HSV was captured at 2000 fps, the simulated signal was downsampled to match that of the HSV. The first 250 ms of the simulated GAW were trimmed to ensure that the numerical model had reached stable oscillations and to avoid any initial numerical transients. This generally resulted in a phase shift between the measured and simulated signals which was corrected by cross correlating the first two cycles of the signals and phase shifting to align the remainder of the signals.

### 4.2. Bayesian Inference

The Bayesian framework for parameter inference seeks a joint probability distribution that represents the probability of all potential values of the parameters of interest, *χ*. Such a density is found through Bayes equation [32]

(13) $$\pi (\chi |y)=\frac{\pi (y|\chi )\pi_{\text{pri}}(\chi )}{\pi (y)}\propto \pi (y|\chi )\pi_{\text{pri}}(\chi ),$$

where *π*(*χ*|**y**) is the posterior probability density function, which contains all probabilistic information about *χ* given observed measurements **y**. The density *π*_pri_(*χ*) is the “prior” probability density, *π*(**y**|*χ*) is the “likelihood”, and *π*(**y**) is the “evidence”. The prior contains known or expected statistical properties of the parameters based on all knowledge available prior to obtaining the measurements. For instance, if subglottal pressure is a model parameter to be inferred, it is known ahead of time that the value cannot be negative, and is likely within a specified bound. The likelihood quantifies the probability of an observed measurement occurring given fixed parameter values; that is, given a particular model with set parameters, what is the likelihood that the measured data would be observed. Lastly, the evidence is a normalization constant that ensures the Law of Total Probability is satisfied.

In the present work, the importance sampling approach is used due to the computational complexity of the model [33]. Such approaches have been successfully used previously for the study of phonation [10,11,34]. The fundamental premise of importance sampling is that certain values of the inputs are more important to the parameter being estimated than others. So a greater weight, which we hereafter refer to as an importance weight, is allocated to those regions in the parameter space that exhibit a better fit to the measurements. In particular, random samples of *χ* are drawn from some proposal distribution, and the resulting observations are simulated using the randomly drawn parameters. The likelihood distribution is then used to probabilistically quantify the goodness of fit to the measured data, from which an importance weight is allocated. When a sufficiently large number of random draws have been computed, a new ensemble is constructed by sampling from the random draws in proportion to their computed importance weight. This can be summarized as follows:

- Initialization: generate an ensemble ${\left\{{\tilde{\chi }}^{\left(l\right)}\right\}}_{l=1}^{N}$ of *N* random samples from the proposal distribution *π*_0_(*χ*).
- Update: for each of the drawn samples, calculate the relative likelihood and normalize to get the importance weight for that sample
  (14) $$w^{\left(l\right)}=\frac{1}{W}\pi \left(y|{\tilde{\chi }}^{\left(l\right)}\right),W=\sum_{l=1}^{N}\pi \left(y|{\tilde{\chi }}^{\left(l\right)}\right).$$
- Resample: generate another ensemble ${\left\{\chi^{\left(l\right)}\right\}}_{l=1}^{N}$ by sampling each ${\tilde{\chi }}^{l}$ with probability *w*^*ℓ*^.

If the proposal distribution is chosen to be the prior density, the final resampled ensemble converges in distribution to the posterior [35]. As a result, the resampled ensemble can then be used to compute sample-based point and spread estimates of the posterior. Herein, the sampling ensembles consist of 50,000 random draws from the prior distributions, which treats each parameter as independent. The sample mean of each ensemble is used to estimate the material properties and the sample standard deviation serves as an uncertainty estimate. The sensitivity of the results to the number of samples is considered in Section 5.3.

The above algorithm defines importance weights in terms of the likelihood density $\pi (y|\tilde{\chi })$, which is determined by the specific error model [33]. In particular, if errors are modeled as unbiased additive normal errors the likelihood density is given by

(15) $$\pi (y|\theta )\propto \text{exp}\left(\frac{-1}{2\sigma_{e}^{2}}{\left\|A_{\text{m}}-A_{\text{s}}\right\|}^{2}\right),$$

where *σ*_*e*_ is the expected standard deviation of the measurement error, and ∥**A**_m_ − **A**_s_∥^2^ is the squared two-norm of the difference between the vectors of the measured and simulated GAWs, respectively. In this work we choose *σ*_*e*_ = 1 mm^2^; this assumes a noise level of around 6%, which is quite large. Such a large noise level was chosen to “whiten” the likelihood to compensate for any model errors that are present [33].

The prior distribution for all estimated parameters were assumed uniform so as to impart the least information into the posterior. Table 2 lists the parameters to be estimated, their experimental “ground truth” values (when known), and the bounds of the uniform prior distribution. The specific bounds for these priors were selected to ensure that the priors sampled a sufficiently wide range of combinations of the parameters and were selected in an ad hoc manner based on an amplification of the expected uncertainty in the experimental value or expectations about that value. We note that a more informative prior distribution (e.g., a Normal distribution) will generally improve estimates and reduce uncertainties, see [36] for more details of how different priors impact estimates.

## 5. Results and Discussion

Considering the case without medial compression as an exemplar, the estimated material properties from the Bayesian inferences are presented in Table 3. Overall, the estimated parameters show good agreement with the “ground truth” experimental values; the maximum discrepancy between the estimates and the experimental values occurs with the Young’s modulus of the ligament with a 9.6% difference. We note that the experimental values, while considered the “ground truth”, are themselves approximate values estimated from the silicone mixture fractions.

We observe that *E*_bdy_ and *E*_cvr_ are over-estimated while *p*_sub_ is under-estimated when compared with the experimental values. The under-estimation of subglottal pressure is likely due to the simplified fluids model being used resulting in high nodal pressures. Over-estimating Young’s modulus of the cover is likely due to the lack of an epithelium in the FE model. The epithelium in the silicone VFs is extremely thin, but has a high Young’s modulus (45 kPa). As a result it is likely that the estimate for *E*_cvr_ is slightly elevated to compensate. It is unclear why the estimate for the body is consistently higher than expected, but may be related with the under-estimation of *E*_lig_. An important observation, however, is that all of the experimental values fall within two standard deviations of the estimated values. This indicates that the use of a FE model of the VFs is statistically capable of inferring accurate estimates of the material properties from a GAW. The relative uncertainty (standard deviation divided by the estimated value) shows that all estimates except for the Young’s modulus of the ligament have uncertainties of approximately 3%; the Young’s modulus of the ligament has an 8.6% level of uncertainty.

The relatively large bias and uncertainty for the ligament stiffness in comparison with the other parameters potentially results from the comparative insensitivity of the FE kinematics to this parameter. The ligament is a small internal region of the geometry and as a result has less impact on the large scale kinematics compared with the body and cover layers. Since *E*_lig_ is the only parameter defined in this region, a range of values are likely to generate similar GAWs, and any error in the estimate of *E*_lig_ can be compensated for by slight adjustments to *E*_bdy_ and/or *E*_cvr_. Despite the modest difficulty in estimating this parameter, overall Bayesian inference is able to accurately estimate the VF material properties from HSV data alone, presuming that the histology of the folds are known a priori.

**Figure 3.**
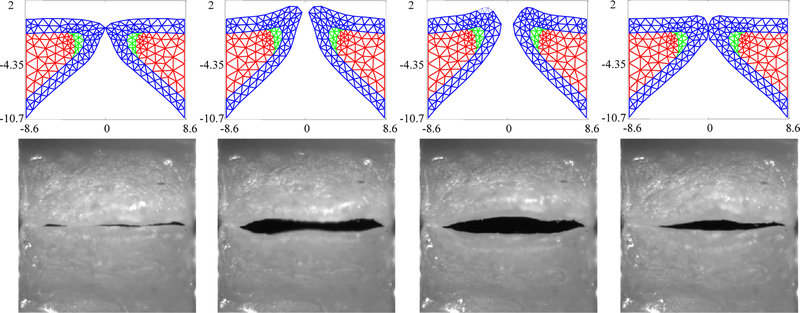
Kinematics of the FE model in comparison with the observed high speed videoendoscopy (HSV) for the case with no medial compression (*p*_sub_ = 1 kPa) at several time points throughout a single oscillation cycle.

Figure 3 compares the kinematics of the FE model employing the estimated material properties from Table 3 with the HSV over a single vibratory cycle. The FE model captures the silicone VF motion well, including the mucosal wave and the pronounced inferior-superior motion of the folds (see the third column in Figure 3). The fourth column highlights that closure of the FE model does not necessarily always correspond with closure of the silicone VFs.

**Figure 4.**
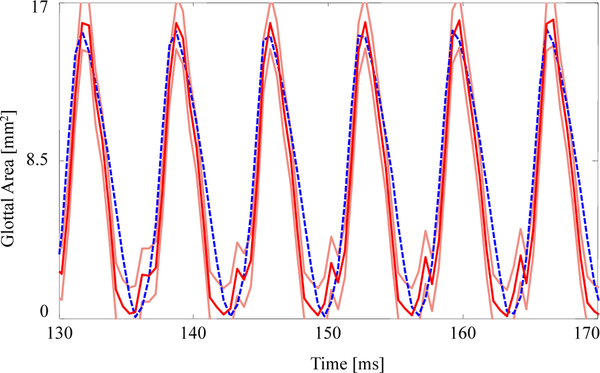
Comparison of the glottal area waveforms extracted from the FE model and the HSV for the case with no medial compression (*p*_sub_ = 1 kPa). Blue dashed line: HSV; red solid line: FE model; orange solid lines: uncertainty bounds from the FE estimate.

The GAW extracted from the FE model and the corresponding HSV for the no medial compression case is presented in Figure 4. As suggested by the fourth column of Figure 3, the FE model closes (GAW reaches zero) before the silicone VFs close. In fact, small openings along the span of the silicone VFs exist during the “closed” phase, as suggested by Figure 2a. Overall, however, the simulated GAW fits the observed data well, with the measured GAW falling within the estimated uncertainty bounds the majority of the time. There are a few persistent mismatches, such as the small peak immediately after opening. Such errors are likely to be model errors induced by approximations, including the use of a simplified 2D approximation of the VFs.

### 5.1. Effect of Medial Compression

As discussed in Section 2.1, HSV was captured with and without medial compression for *p*_sub_ = 1 kPa. Since it was difficult to measure the degree of medial compression experimentally, the actual pre-stress is highly uncertain. As such, the initial position parameter *x*_0_ in the FE model is included as an estimated parameter for both cases.

Table 4 presents the estimated material properties for both medial compression cases. The estimated properties (excepting *x*_0_) agree well with each other and with the “ground truth” values. This is encouraging in two respects: the same silicone VFs were used in both cases, and the pre-stress in the medial compression case is captured in *x*_0_ and thus does not appreciably bias the stiffness estimates. However, the higher parameter uncertainties for the medial compression case is very likely due to the fact that the pre-stress associated with medial compression can be approximated by varying other stiffness parameters. This yields more overall uncertainty in the results, as other parameter combinations can explain the observed data.

The estimates for the initial position parameter, *x*_0_, are statistically different between the two cases. When there is no medial compression the initial position is estimated to be 8.39 mm (8.4 mm corresponds to zero compression, see Figure 2a); with compression, *x*_0_ is estimated to be 8.27 mm. This 0.12 mm difference in the estimates is more than double the sum of the two estimated standard deviations, with a t-value of 3.67, indicating that the FE model was capable of distinguishing between the experimental configurations. This does differ from the 0.4 mm shim placed experimentally to produce the medial compression, though again, the actual degree of experimental medial compression was very difficult to ascertain.

Comparing the case without medial compression with the results in Table 3 shows that the uncertainties in the estimated parameters are larger in the present case despite using the same observation data. By including the extra fitting parameter, *x*_0_, the estimated uncertainties increase due to the higher dimensionality (more parameters being fit given the same input data). That is, with the addition of *x*_0_ as a parameter, there is now an alternative pathway to influence the energy in the system. That said, the uncertainties in both estimates are large enough and the estimated values are similar enough that the two data sets cannot be distinguished statistically.

**Figure 5.**
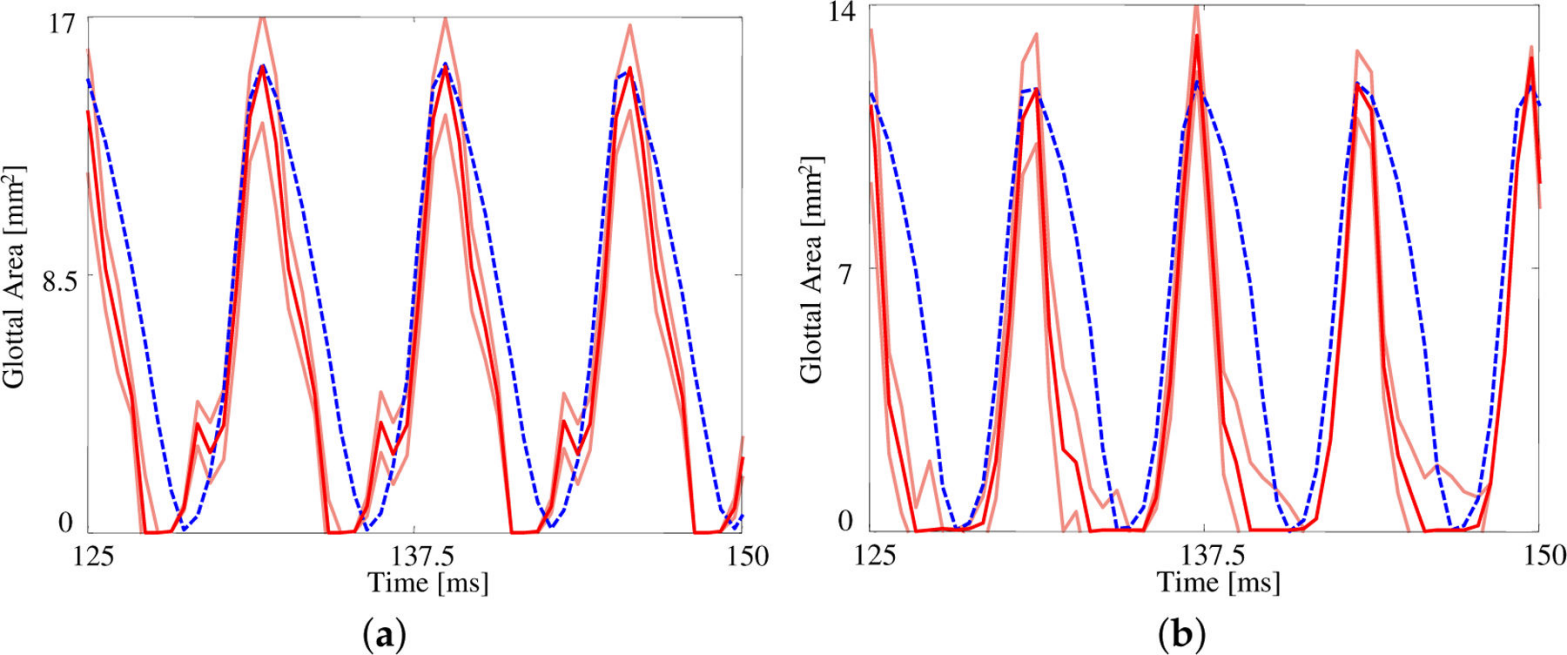
The glottal area waveform extracted from HSV of self-oscillating silicone vocal folds at *p*_sub_ = 1.00 kPa (**a**) without medial compression and (**b**) with medial compression. Blue dashed line: HSV; red solid line: FE model; orange solid lines: uncertainty bounds from the FE estimate.

Figure 5 presents the measured and estimated GAWs for the two medial compression cases, wherein the FE models employ the material properties given in Table 4. Both estimates fit the data reasonably well given the simplified FE model being used. The open quotient and speed quotients are both lower for the FE models, with the effect more pronounced for the case with medial compression. This results in a more peaked GAW in comparison with the HSV. The case without medial compression exhibits larger maximum glottal area, however the maximum contact pressure experienced during collision in the case with compression is 18% higher, on average, due to the pre-stress of the system (825.7 ± 58.6 Pa versus 665.0 ± 53.1 Pa).

### 5.2. Distinguishing between Model Configurations

As the eventual goal of this research is patient-specific modeling, we wish to investigate whether the FE model is sufficiently sensitive and if HSV data provide enough information to distinguish between similar experimental configurations. As a first order exploration we consider varying subglottal pressures for silicone VFs with medial compression. As discussed in Section 2.1, the pressures considered range from 0.91 to 1.18 kPa.

**Figure 6.**
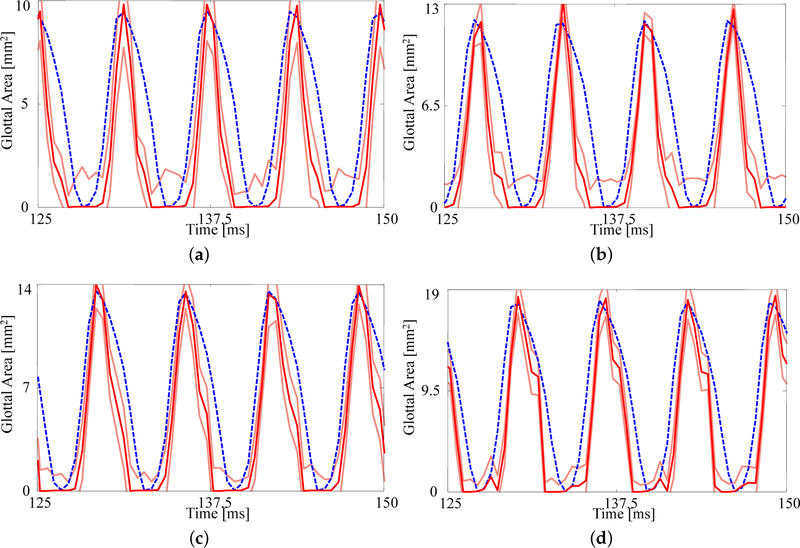
Comparison on the glottal area waveforms extracted from HSV and the FE predictions from the fitted material properties for (**a**) *p*_sub_ = 0.91, kPa (**b**) *p*_sub_ = 1.00 kPa, (**c**) *p*_sub_ = 1.09 kPa, and (**d**) *p*_sub_ = 1.18 kPa. Blue dashed line: HSV; red solid line: FE model; orange solid lines: uncertainty bounds from the FE estimate.

The fits to each GAW are shown in Figure 6 and the resulting estimates of the degree of medial compression, along with the other material properties, are shown in Table 5. Similar to Figure 5, the fits are again reasonable given the simplified model being used. As the subglottal pressure increases, so too does the maximum glottal area, as expected; this increase is captured by the FE model. As with the previous comparison, the open quotient and speed quotient are both lower for the FE model, though the difference decreases with increasing subglottal pressure.

As shown in Table 5, all experimental values fall within two standard deviations of the estimated values; furthermore, the viscosity is estimated to be approximately 3 Poise, which is consistent with the previous results. The estimated medial compression, *x*_0_, varies somewhat from case to case, but all values are within two standard deviations of one another, indicating statistical consistency. Overall, comparing the estimates in Tables 3–5, we find that the estimated material properties are quite consistent across all cases studied, engendering confidence in the method.

Considering the uncertainty in the estimates, we see that as the subglottal pressure increases its uncertainty decreases. As the variance of the measurement noise is treated as fixed at 1 mm^2^ in all estimates, this decrease in uncertainty is not due to a decrease in measurement uncertainty. Furthermore, the same prior distributions are used in all cases. Hence, this decrease in uncertainty is due to an increase in sensitivity of the model, which could be due to the larger glottal width having fewer parameter combinations that are capable of matching the data. Alternatively, the change in uncertainty could be due to the pre-stress model. The estimates computed for *p*_sub_ = 1.09 kPa and *p*_sub_ = 1.18 kPa have *x*_0_ > 8.3 mm, whereas the other two estimates have *x*_0_ < 8.3 mm; in addition, there is a marked decrease in the uncertainties in *x*_0_ as *p*_sub_ increases. This could indicate that a higher level of VF compression results in a model with lower sensitivity to the parameters. That is, the dynamics may be more influenced by pre-stress at higher subglottal pressures.

Overall, the consistency in the material property estimates and the reasonably low relative uncertainty in *p*_sub_ indicates that the FE model is capable of distinguishing between operational conditions. In pairwise t-tests of the four estimates only two pairs fail to reject the hypothesis of unique data sets (95% confidence); those pairs are *p*_sub_ = 0.91 kPa versus 1.00 kPa and *p*_sub_ = 1.00 kPa versus 1.09 kPa. In these two pairs there are small changes in the experimental subglottal pressures (0.09 kPa) and similar fundamental frequencies. Interestingly, there is significant difference between the two cases with the highest subglottal pressures, despite also only differing by 0.09 kPa in line with the decreased uncertainty in subglottal pressure at these conditions. We note there is a more marked difference between fundamental frequencies in these cases.

For further validation we compare the volumetric flow rate, *Q*, estimated from the model with the experimentally measured values; volumetric flow rate was not included in the estimation process and thus provides an independent measure for method/model validation. Table 6 compares the estimated and experimentally measured values. The estimated flow rate is derived directly from the Bernoulli flow model embedded in the FE model (see Section 3) as $Q(t)=1.3A_{\text{min}}(t)\sqrt{2\varrho^{-1}\left(p_{\text{sub}}-p_{\text{sup}}\right)}$ where *ϱ* = 1.14 kg m^−3^ is the density of air. As can be seen in the table, the estimated values generally agree well with, but tend to slightly over-predict, the experimentally measured values. Excepting for the case of *p*_sub_ = 1.18 kPa, the estimated values are all within one standard deviation of the measurements.

With the FE models developed for the four cases and validated with an independent measure (*Q*), an additional parameter is explored that is not available experimentally. Table 7 presents the average mean and maximum contact pressures experienced during collisions for the four subglottal pressures. The contact pressures increase with increasing subglottal pressure, which qualitatively agrees with previous studies [37]. Excepting for the *p*_sub_ = 1.18 kPa case, the contact pressures are all less than the subglottal pressure. The silicone VFs employed in this study qualitatively do not appear to have vigorous contact when self-oscillating, and as such, the contact pressures may indeed be less than the subglottal pressure. The outlier in the estimated pressure data is the highest subglottal pressure, which exhibits mean and maximum contact pressures well above the subglottal pressure. This is also the case that predicted a volumetric flow rate well above the measured value, suggesting that this model is less reliable. Interestingly, this is the case that had the lowest material property uncertainties. It is likely that the poor agreement in *Q*, and the exceedingly high contact pressures for this case, are a result of more complex vibratory patterns for the silicone VFs at this higher subglottal pressure that are not captured well with the simplified 2D FE model. This suggests that additional measures may be required in the estimation process to generate an accurate model for this case.

### 5.3. Sensitivity Analysis

The estimates presented in Section 5 were produced for fixed ensemble size for the importance sampling, and fixed time step size and mesh density for the FE model. These parameters influence the quality of the numerical model and the estimation procedure while also impacting the computational load. The estimates presented in the previous sections, for example, took 140 h for 16 parallel threads on a AMD Ryzen Threadripper 1950X with 16 cores at 3.4 GHz and 128 GB of RAM to run the importance sampling for all 50,000 samples. As such, there is motivation to use a coarser model (larger/fewer elements and larger time step) and fewer samples to decrease the computational load. To ensure that the estimates presented in this work are not conditional on the numerical parameters being used in the model we explore the sensitivity of the results to them in this section. We use the case without medial compression, again, as the exemplar.

#### 5.3.1. Ensemble Size

**Figure 7.**
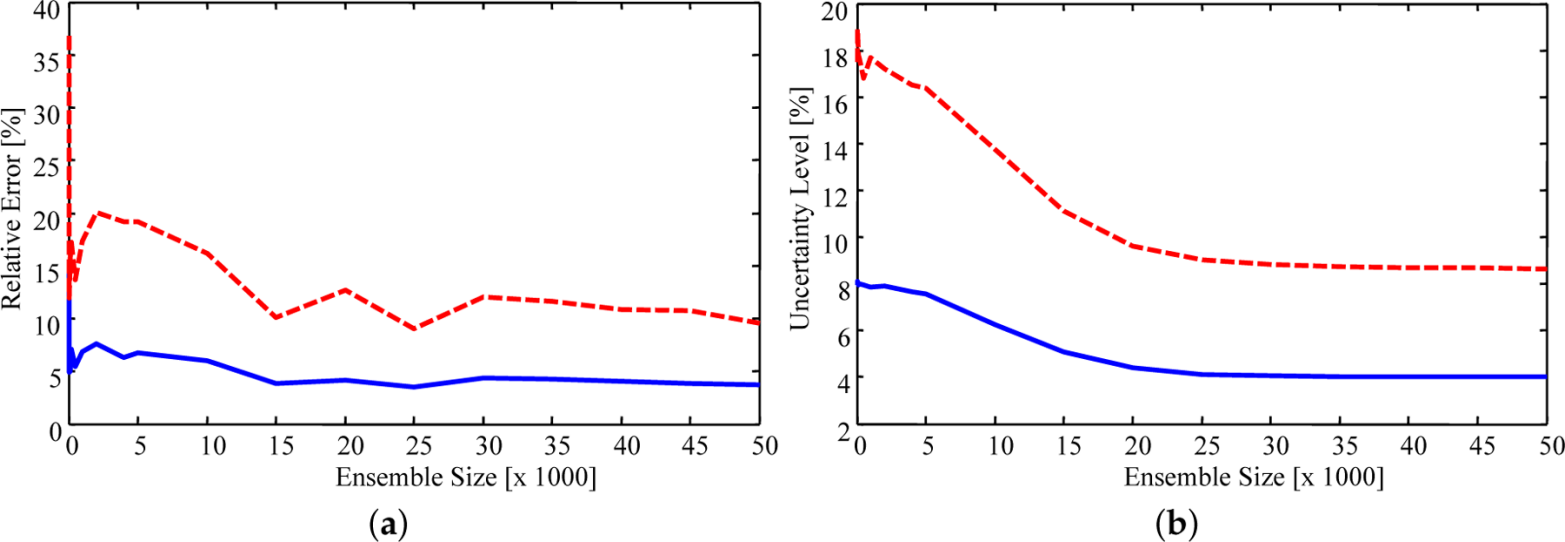
(**a**) Relative error of the estimates; and (**b**) relative uncertainty for increasing ensemble size. Solid blue line: average; dashed red line: maximum.

Sensitivity of the results to the importance sampling ensemble size was checked by computing estimates with a progressively smaller number of samples from the priors. Figure 7 presents the relative error, defined as the percentage difference in the estimate and experimental value, and uncertainty level for ensemble sizes ranging from 100 to 50,000. It was found that, on average, the estimated values stabilize as the ensemble size increases. The peaks which occur at approximately 15,000 and 25,000 samples are due to the sample-based nature of the estimate. Since the estimates are the sample mean of the resampled ensemble the exact estimated value for each parameter changes as more samples are included in the ensemble. As such, it is encouraging to note that the estimates do appear to stabilize. Specifically, the average error of the estimates stabilizes when using an ensemble size of approximately 15,000, with the maximum error stabilizing with approximately 40,000 samples. The uncertainty smoothly decreases as the ensemble increases, stabilizing when approximately 25,000 samples are used. Error in the estimates converges faster than the uncertainty since an accurate estimate of the mean is easier to attain than a stable estimate of the variance.

#### 5.3.2. FE Time Step and Triangulation

Estimates were produced using time step sizes of *h* = 0.025, 0.05, and 0.1 ms and triangulations with 172, 205, and 263 elements over the same geometry. For direct comparison, the estimates were computed using an importance sampling ensemble size of 50,000.

**Figure 8.**
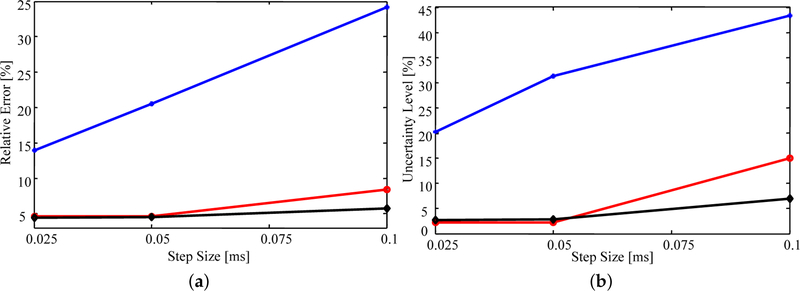
The average (**a**) relative error and (**b**) relative uncertainty of the estimates as a function of time step size for various triangulations. Blue with the plus markers: 172 elements; red with circle markers: 205 elements; black with diamond markers: 263 elements.

Figure 8a shows that, on average, the relative error (as defined above) in the estimates is very similar whether the triangulation involves 263 or 205 elements. In fact, all estimated parameter values differ by 0.1% or less for the two triangulations. This difference is likely due to numerical error resulting from the sample-based nature of importance sampling. In contrast, the lower density mesh introduces a larger error for all step sizes, but rapidly improves with decreasing step size.

Similar trends are observed in Figure 8b where decreasing step size or increasing mesh density results in a decrease in uncertainty. The observed decrease in uncertainty with decreasing time step and increasing mesh density results from having a higher fidelity numerical model that is more sensitive to the parameter values; that is, small changes in the parameter values will have a larger impact on the simulated data [33]. As a result, the uncertainty of the estimates will decrease as the fidelity of the model increases. However, as the time step decreases and mesh density increases the computational cost grows exponentially. Thus, examining how the estimated uncertainty behaves as the fidelity of the model increases becomes quickly infeasible.

## 6. Conclusions

To date, the approaches employed for developing subject-specific numerical VF models have focused on lumped-elements for the fitting model in the inverse analysis; as such, parameter estimates are often greatly abstracted from the physical tissue properties. To overcome these limitations, the present work proposes a FE model of the VFs for a fitting model. The FE model captures the geometry and layered structure of the VFs more accurately, treating them as a multi-layered viscoelastic body, thus better approximating their kinematics. Since the FE model directly employs the tissue properties, such as Young’s moduli, these properties are estimated directly. Estimation of material properties was demonstrated using HSV data of silicone VFs as the observation, showing good agreement between the estimated and “ground truth” material properties.

The robustness of the method was demonstrated by considering experimental data with different degrees of medial compression and differing subglottal pressures. The FE model faithfully recovered the material properties in all cases, including the degree of medial compression, which was embedded into the FE model in the form of base displacement. This suggests that the employed Bayesian framework using a FE fitting model is sufficiently sensitive to distinguish between different experimental conditions, even though the model was restricted to two dimensions.

The FE models were validated by comparing the volumetric flow rate predicted by the model with experimentally measured values. This observation was not included in the estimation process and, as such, was an independent measure. The volumetric flow rate was slightly over-predicted, but generally agreed well. The exception was the highest subglottal pressure case, which was considerably over-predicted. Additionally, the contact pressures extracted from the developed FE models were found to increase with increasing subglottal pressure and medial compression, which is a trend that qualitatively agrees with previous studies. The highest subglottal pressure case was again an outlier, suggesting that the FE model for that case does not accurately capture the kinematics of the silicone VFs, likely due to the 2D geometry. This could potentially be improved by incorporating additional observations in the estimation procedure or expanding to a three-dimensional model.

The stability of the results was examined with respect to numerical parameters, such as the importance sampling ensemble size, time step size, and mesh density. Estimated values converged at relatively modest ensemble sizes, though resolving the uncertainties required considerably more samples. Decreasing the time step size and increasing the mesh density lead to smaller uncertainties at the cost of significant computational time. One of the main drawbacks to our proposed model is the computational complexity; this cost will increase if more complex fluids models, a three-dimensional geometry, or acoustics are included.

One source of uncertainty that has not been considered in this work is the structure of the layers. All estimates in this work have been computed with a FE model that was formed treating the layers and dimensions of the silicone VFs as perfectly known. The use of imperfect layers will affect the estimates and uncertainties of the material properties, however, this is the subject of ongoing research and requires a further examination.

As an introductory effort, incorporating a FE fitting model into the Bayesian estimation framework has shown good promise. Future work includes validating the contact pressure estimates with experimental data, implementing a three-dimensional FE model, and employing clinical HSV.

## Figures and Tables

**Table 1.** Different mixture ratios of silicone and their corresponding Young’s moduli. A ratio of 1:1:x means that the layer was formed by mixing 1 part A, 1 part B, and x parts thinner by weight.

VF Layer	Material	Ratio (A-B-Thinner)	Young’s Modulus (kPa)
Body	Ecoflex	1-1-1	11.8
Ligament	Ecoflex	1-1-4	2
SLP	Ecoflex	1-1-6	0.6
Epithelium	Dragon Skin	1-1-1	45

**Table 2.** List of the the experimental values and prior distributions used for the estimated parameters. The distribution bounds are the bounds of the employed uniform prior distribution (lower bound, upper bound).

	*E*_bdy_ (kPa)	*E*_cvr_ (Pa)	*E*_lig_ (kPa)	*p*_sub_ (Pa)	*ρ* (kg/m^3^)	*η* (Poise)	*x*_0_ (mm)
Experimental Value	11.8	600	2	1000	1049.75	-	-
Distribution Bounds	(9, 15)	(250, 950)	(0.5, 3)	(400, 1800)	(950, 1200)	(1, 7)	(7.4, 8.4)

**Table 3.** Material property estimates for the case without medial compression.

	*E*_bdy_(kPa)	*E*_cvr_(Pa)	*E*_lig_(kPa)	*p*_sub_ (Pa)	*ρ* (kg/m^3^)	*η* (Poise)
Experimental Value	11.8	600	2	1000	1049	-
Estimated Value	12.15	636.2	1.808	989.9	1051	3.079
Standard Deviation as a percentage of the Estimate	3.19%	3.35%	8.63%	3.21%	0.31%	3.48%

**Table 4.** Estimates and the associated uncertainties as a percentage of the estimate values (in brackets) for material properties of the silicone vocal folds and initial position for cases with and without medial compression (*p*_sub_ = 1 kPa).

Data Set	*E*_bdy_ (kPa)	*E*_cvr_ (Pa)	*E*_lig_ (kPa)	*p*_sub_ (Pa)	*ρ* (kg/m^3^)	*η* (Poise)	*x*_0_ (mm)
Experimental Value	11.8	600	2	1,000	1049	-	-
Without Medial Compression	12.32 (3.96%)	632.1 (3.53%)	1.807 (9.74%)	987.7 (3.98%)	1051 (0.25%)	2.981 (4.66%)	8.39 (0.38%)
With Medial Compression	12.28 (4.30%)	629.8 (4.1%)	1.842 (12.16%)	991.9 (4.536%)	1051 (0.26%)	3.11 (4.66%)	8.27 (0.30%)

**Table 5.** Material properties estimates for varying subglottal pressures with medial compression. The associated estimate uncertainties as a percentage of the estimate values are in brackets.

*p*_sub_ (kPa)	*E*_bdy_ (kPa)	*E*_cvr_ (Pa)	*E*_lig_ (kPa)	*p*_sub_ (Pa)	*ρ* (kg/m^3^)	*η* (Poise)	*x*_0_ (mm)
0.91	12.38 (4.31%)	643.0 (6.22%)	2.375 (12.67%)	901.2 (6.16%)	1048 (0.28%)	2.98 (6.28%)	8.29 (0.37%)
1.00	12.28 (4.3%)	629.8 (4.1%)	1.842 (12.16%)	991.9 (4.53%)	1051 (0.26%)	3.11 (4.66%)	8.27 (0.30%)
1.09	12.15 (2.27%)	635.1 (3.01%)	1.858 (10.39%)	1062 (2.89%)	1047 (0.16%)	3.01 (4.45%)	8.33 (0.28%)
1.18	12.25 (2.07%)	639.6 (2.74%)	2.098 (8.1%)	1164 (2.43%)	1049 (0.15%)	3.03 (3.86%)	8.32 (0.22%)

**Table 6.** Experimental and estimated mean volumetric flow rates along with a measure of uncertainty in the form of a standard deviation as a percentage of the estimated value. Data are all in mL/s.

*p*_sub_ (kPa)	0.91	1.00	1.09	1.18
Experimental	236	260	291	307
Estimated	242.5	272.2	301.4	361.6
Uncertainty	8.29%	7.02%	*t*6.14%	4.87%

**Table 7.** Estimated average maximum and mean contact pressures along with a measure of uncertainty in the form of a standard deviation as a percentage of the mean estimated value for the cases with medial compression. All data are in (Pa).

*p*_sub_ (kPa)	0.91	1.00	1.09	1.18
Maximum	405.8	825.7	1032.8	2000.9
Mean	246.5	475.8	662.6	1469.8
Uncertainty	13.87%	12.32%	10.14%	6.67%
